# Enhancing Performance of Breast Ultrasound in Opportunistic Screening Women by a Deep Learning-Based System: A Multicenter Prospective Study

**DOI:** 10.3389/fonc.2022.804632

**Published:** 2022-02-10

**Authors:** Chenyang Zhao, Mengsu Xiao, Li Ma, Xinhua Ye, Jing Deng, Ligang Cui, Fajin Guo, Min Wu, Baoming Luo, Qin Chen, Wu Chen, Jun Guo, Qian Li, Qing Zhang, Jianchu Li, Yuxin Jiang, Qingli Zhu

**Affiliations:** ^1^Department of Ultrasound, Chinese Academy of Medical Sciences and Peking Union Medical College Hospital, Chinese Academy of Medical Sciences and Peking Union Medical College, Beijing, China; ^2^Department of Ultrasound, First Affiliated Hospital, Nanjing Medical University, Nanjing, China; ^3^Department of Ultrasound, Peking University Third Hospital, Beijing, China; ^4^Department of Ultrasound, Beijing Hospital, Beijing, China; ^5^Department of Ultrasound, Nanjing Drum Tower Hospital, Nanjing, China; ^6^Department of Ultrasound, Sun Yat-sen Memorial Hospital, Guangzhou, China; ^7^Department of Ultrasound, Sichuan Provincial People’s Hospital, University of Electronic Science and Technology of China, Chengdu, China; ^8^Department of Ultrasound, First Hospital of Shanxi Medical University, Taiyuan, China; ^9^Department of Ultrasound, Aero Space Central Hospital, Beijing, China; ^10^Department of Ultrasound, Henan Provincial Cancer Hospital, Zhengzhou, China

**Keywords:** breast cancer, ultrasound, deep learning, computer-aided diagnosis, elastography

## Abstract

**Purpose:**

To validate the feasibility of S-Detect, an ultrasound computer-aided diagnosis (CAD) system using deep learning, in enhancing the diagnostic performance of breast ultrasound (US) for patients with opportunistic screening-detected breast lesions.

**Methods:**

Nine medical centers throughout China participated in this prospective study. Asymptomatic patients with US-detected breast masses were enrolled and received conventional US, S-Detect, and strain elastography subsequently. The final pathological results are referred to as the gold standard for classifying breast mass. The diagnostic performances of the three methods and the combination of S-Detect and elastography were evaluated and compared, including sensitivity, specificity, and area under the receiver operating characteristics (AUC) curve. We also compared the diagnostic performances of S-Detect among different study sites.

**Results:**

A total of 757 patients were enrolled, including 460 benign and 297 malignant cases. S-Detect exhibited significantly higher AUC and specificity than conventional US (AUC, S-Detect 0.83 [0.80–0.85] vs. US 0.74 [0.70–0.77], *p* < 0.0001; specificity, S-Detect 74.35% [70.10%–78.28%] vs. US 54.13% [51.42%–60.29%], *p* < 0.0001), with no decrease in sensitivity. In comparison to that of S-Detect alone, the AUC value significantly was enhanced after combining elastography and S-Detect (0.87 [0.84–0.90]), without compromising specificity (73.93% [68.60%–78.78%]). Significant differences in the S-Detect’s performance were also observed across different study sites (AUC of S-Detect in Groups 1–4: 0.89 [0.84–0.93], 0.84 [0.77–0.89], 0.85 [0.76–0.92], 0.75 [0.69–0.80]; *p* [1 vs. 4] < 0.0001, *p* [2 vs. 4] = 0.0165, *p* [3 vs. 4] = 0.0157).

**Conclusions:**

Compared with the conventional US, S-Detect presented higher overall accuracy and specificity. After S-Detect and strain elastography were combined, the performance could be further enhanced. The performances of S-Detect also varied among different centers.

## Introduction

A dramatic increase in breast cancer incidence was reported in China in recent years and early detection is essential to reduce the mortality of breast cancer (1). Different from western countries, in which mammography is the most used method for breast screening, in China, mammography is not so popular due to its relatively low accuracy for women with dense breasts, which accounts for more Chinese women than Caucasian women (2), as well as the inaccessibility of the equipment in some regions of the country. Ultrasound (US) has become the most common method for screening breast cancer in China, due to its high detection rate of breast cancers in dense breast tissue and convenience (3, 4). A multicenter study of the country revealed a better diagnostic performance and higher cost efficiency of US than that of mammography in breast screening, and US screening has been recommended for high-risk women by a nationwide guideline (5–7). To note, US screening is often opportunistic in China due to different economic statuses and the insurance policies of different areas.

Despite the good performance, there still exist several drawbacks of breast US. In consideration of its widespread use in China, it is imperative to enhance the diagnostic performance of US. Moreover, US tends to present a high sensitivity in detecting malignant lesions but a relatively low positive predictive value (PPV), causing unnecessary biopsies or repeated examinations in short intervals (8). Usually, the category 4 and 5 lesions of the Breast Imaging Reporting and Data System (BI-RADS) lexicon identified by screening US are strongly recommended for further evaluation. But in clinical practice, patients with screening-detected BI-RADS 3 lesions also tend to choose a second-time US examination or biopsies, resulting in a high recall rate and false-positive results (9). Moreover, the operator dependence of breast US also has an adverse impact on the screening results (10, 11). Hence, new imaging techniques that can overcome these defects of US can be of great clinical value.

Computer-aided diagnosis (CAD) systems, which are designed to help doctors in diagnosing diseases to provide automatic segmentation or diagnosis of medical images (12), has been intensively investigated in the field of breast imaging in recent years, especially the systems constructed on deep learning (DL) method (13–16). S-Detect™ is one of the DL-based CAD programs for classifying breast lesions through US images. It is an onboard software integrated on a commercial US machine. The software is composed of a DL algorithm, which has been trained by a large number of ultrasonic images of breast lesions. When provided with a static US figure showing a suspicious breast lesion, the software can give a dichotomic diagnosis of the lesion, as possibly benign or possibly malignant. Several studies from Europe and Asia have validated its excellent performance in enhancing the diagnostic accuracy of US by increasing the specificity, consequently assisting in reducing unnecessary biopsies of breast lesions (17–20). According to our preliminary single-center research, S-Detect™ can provide a reliable classification for the asymptomatic screening-detected breast lesions (21). In order to further investigate its benefit for those asymptomatic patients with US screening-detected breast lesions, we launched this nationwide multicenter study about the clinical use of S-Detect™ in China. In this study, patients with opportunistic screening-detected breast lesions who were going to receive a second-time breast US examination were enrolled and evaluated by the new CAD technique. As far as we know, this is the first multicenter study about S-Detect™, and none of the previous studies have investigated the feasibility of the software for US screening-detected breast lesions.

Apart from utilizing the CAD system alone, we also investigated the role of combining the CAD technique and elastography in promoting the diagnostic efficacy of US in re-evaluating opportunistic US screening-detected breast lesions. Elastography is applied as a complementary for US to characterize breast lesions by providing information about tissue stiffness (22–24). For strain elastography, compressive force is implemented on breast tissues, and the tissue stiffness is often expressed as pseudo-color mapping or fat-to-lesion strain ratio (SR), both of which have been verified as an effective method to present the elasticity of tumor tissues and help increase accuracy and specificity of diagnosing breast cancers (25, 26). Recently, a newly developed built-in software of strain elastography, E-breast™, has been put into clinical use and distinguished with its ability in providing a relative objective value of SR. Considering that both S-Detect™ and E-breast™ can provide relatively objective imaging parameters for breast US, it will be of interest to explore the potential value of combining the use of the two novel imaging methods. Therefore, in this multicenter study, we also evaluated the diagnostic performance of the combination of S-Detect™ and E-breast™ in diagnosing breast lesions and investigate the clinical value of the combination. Therefore, in this multicenter study, we aim to investigate the feasibility of S-Detect™, a DL CAD tool for breast US, and its combination with elastography in diagnosing breast cancer for patients with opportunistic screening-detected breast lesions. We also compared the diagnostic performances of S-Detect™ among different centers.

## Materials and Methods

This study was designed as a prospective multicenter one, and it was approved by the institutional review board of all of the participating centers. Written informed consent was signed by each recruited patient. A total of 9 medical centers from six provinces and municipalities were involved in this study. All the centers are general hospitals and own large-scale breast imaging departments, where US is performed for patients with breast lesions as a clinical routine. Before the inception of the study, we enacted a protocol regulating standards for image and clinical data acquisition, operation method for the software, and classification criteria for enrolled patients and lesions. The investigators of these medical centers received training on the protocol and participated in the study after fully understanding the protocol and breast US knowledge. The study has also been registered at ClinicalTrials.gov (NCT03851497).

### Patient Recruitment

From January 2019 to December 2019, a total of 757 patients from the medical centers were consecutively recruited in this study. Asymptomatic female patients with breast masses from the participated hospitals were enrolled in this study. Before participation, those patients were found to have BI-RADS 3–5 breast masses by bilateral breast US screening within 3 months and referred to the medical centers for further diagnostic imaging tests.

The definitions for asymptomatic individuals are listed as follows.

1. no self-palpable breast masses
2. no severe breast pain that could not be explained by physiological reason
3. no nipple discharge
4. no changes in breast appearance, including nipple inversion, skin redness, and skin retraction

Exclusion criteria included breast malignancy history, pregnancy, lactation, and refusal to participate in the study. When more than one lesion was found eligible in a patient, we selected the suspicious lesions or the largest ones. The patients received biopsies after US examinations within 2 weeks and had final pathological results referred to as the gold standard for classifying breast mass.

### Imaging Analysis

#### Conventional Breast Ultrasound Examinations and Image Acquisition

The radiologists in this study who performed US examinations had at least 5-year experience in breast US. In all medical centers, the radiologists performed US examinations with a high-frequency linear transducer (L3-12), under the breast preset on the US machine (RS80A, Samsung Medison Co., Ltd., Korea) according to standard scanning protocol. For the grayscale US, the focal zone was adjusted with the lesion depth, and the gain was set at 25%–50%. For color Doppler, the imaging settings included a scale of 3 cm/s, a wall filter of 50–100 Hz, and a rectangular sampling box with no angulation. After detection of the target lesion, conventional grayscale US and color Doppler US were consecutively performed on two orthogonal planes. The radiologists assessed the lesions after the dynamic scanning. The image on the largest diameter of the lesion was recorded for further reading and CAD analysis by the radiologists.

#### Strain Elastography

The built-in software of strain elastography, E-breast™ (Samsung Healthcare, Seoul, South Korea), was utilized in this study. After the acquisition of elastographic imaging of a breast lesion, the SR between the mass and surrounding fat can be calculated using E-breast™. Elastography was performed by the same radiologist after completing a conventional breast US examination. Elastography imaging was acquired with freehand compression. Imaging methods have been previously described in detail (22, 26). Briefly, the radiologist put the probe perpendicular to the chest wall and parallel to the pectoralis muscle and applied the probe with only light pressure. The proper pressure was gauged under the guidance of a compression guide bar to acquire appropriate images for analysis. The compression guide bar was on the right side of the working interface of E-breast™ to guide the operators in applying compressive force. The compression guide bar displayed the degree of pressure in colors between 0 and 7 stages. 0 stage (all black) represented no movement of the probe; 1–2 stage (gray) represented not enough compression speed; and 3–7 stage (green) represented moderate compression speed, indicating a good-quality strain image. When the guide bar reached the 3–7 stage, the strain image was regarded as qualified for further analysis and selected for calculation subsequently.

For calculating SR, one elliptical frame for sampling region of interest (ROI) was placed on the target lesion on the elastographic image, and the straining value on the fat area was provided automatically by the software (22).


$$SR=\frac{\text{Average fat strain (automatically derived)}}{\text{Average lesion strain (lesion ROI)}}$$

Elastography was performed three times for each patient. The maximal SR was used for final analyses. The same depth, focus, and gain parameters were employed for elastography as were used for conventional imaging.

#### S-Detect™ Classifications for Breast Lesions

S-Detect™ (Samsung Healthcare, Seoul, South Korea) was embedded in the RS80A US system, and the radiologists opened the working interface of S-Detect™ after finishing giving a diagnosis of the lesions. The slice with the maximal size of the lesion was recorded by the radiologists performing US examinations for S-Detect™ analysis. The grouping for breast lesions of S-Detect™ was performed automatically after clicking the center of the lesion on a grayscale slice, presenting dichotomic results as possibly benign and possibly malignant by the software, along with automatically recognized ultrasonographic features, including shape, orientation, margins, pattern, and posterior acoustic features.

### Image Interpretation

Before performing elastography and the CAD system, the radiologists gave a diagnosis of the lesion based on the BI-RADS lexicon on site (27). The lesions were classified into BI-RADS 3, 4, and 5 according to their ultrasonographic features, and the results of elastography and S-Detect had no impact on the diagnosis of radiologists. The BI-RADS classification of each lesion was decided by the US-operating radiologists after identifying the aforementioned US features. A cutoff value was set at category 4 to transform BI-RADS classification into a dichotomic form. Category 3 lesions were allocated to possibly benign, and categories 4 and 5 were put as possibly malignant.

### Statistical Analysis

Previous studies showed that S-Detect could increase AUC from 0.76 to 0.83 (17–20). By applying that the disease prevalence of 10%, 90% power, 5% two-sided significance, and 10% missing data, a sample size of 768, including 192 malignancies and 576 benign, was figured out for this multicenter study.

A series of statistical parameters pertaining to the diagnostic performance of a test were calculated, including sensitivity, specificity, positive likelihood ratio (PLR), negative likelihood ratio (NLR), PPV, negative predictive value (NPV), receiver operating characteristic (ROC) curve, and area under the ROC curve (AUC) (28). The optimal cutoffs of SR were also calculated using ROC analysis, defined as the closest point on the ROC curve to the point (0, 1). We used 2 × 2 contingency tables, a chi-square test for comparing sensitivity and positivity, a generalized estimating equation for comparing PPV, and the method proposed by DeLong et al. for comparing AUC values (29). A *p*-value of <0.05 was considered statistically significant. A forward stepwise logistic regression method was applied to combine S-Detect™ and strain elastography. We regarded the result of S-Detect as categorical data and SR as continuous variables to construct the model. An equation was acquired subsequently after regression. We presented the equation representing the combination of the two methods determined by the multiple regression method in the form of a nomogram. The model underlying the nomogram was to classify breast lesions based on the results of S-Detect and strain elastography.

Then we divided the nine medical centers into four groups (Groups 1–4), on the basis of local economic and medical service resources of the geographical regions with different breast cancer incidence. Group 1 and Group 2 were the centers located in Beijing and the east area of China, respectively, both of which were highly developed regions of China. Group 3 and Group 4 were the centers in less-developed regions, including the west and central regions of China, respectively. Compared with Groups 3 and 4, Groups 1 and 2 are located in regions with better economic status and a higher level of medical care. In China, the incidence rate of breast cancer is higher in socioeconomically developed coastal cities, with the highest age-standardized rate (ASR) of 46.6 cases/100,000 women. In contrast, in less developed areas of the central and western regions, the ASR for breast cancer can be less than 7.94 cases per 100,000 women (30). In general, the incidence rates of breast cancer in the regions of Groups 1 and 2 were higher than those of Groups 3 and 4. The diagnostic performances of conventional US and S-Detect of the four groups were calculated and compared, respectively. The AUC values of the four groups, representing the overall accuracy, were compared. The AUC values of S-Detect in different regions or medical centers were also compared using the method described by Hanley and McNeil (31) for comparing the AUC of two independent ROC curves. We compared the sensitivity and specificity among different groups using the Mann–Whitney test of the Normal approximation in independent samples (32).

Statistical analysis was performed using Medcalc (MedCalc software, version 15, Ghent, Belgium), R (http://www.R-project.org), and EmpowerStats software (X&Y Solutions).

## Results

### Basic Characteristics of Enrolled Patients

**Figure 1:**
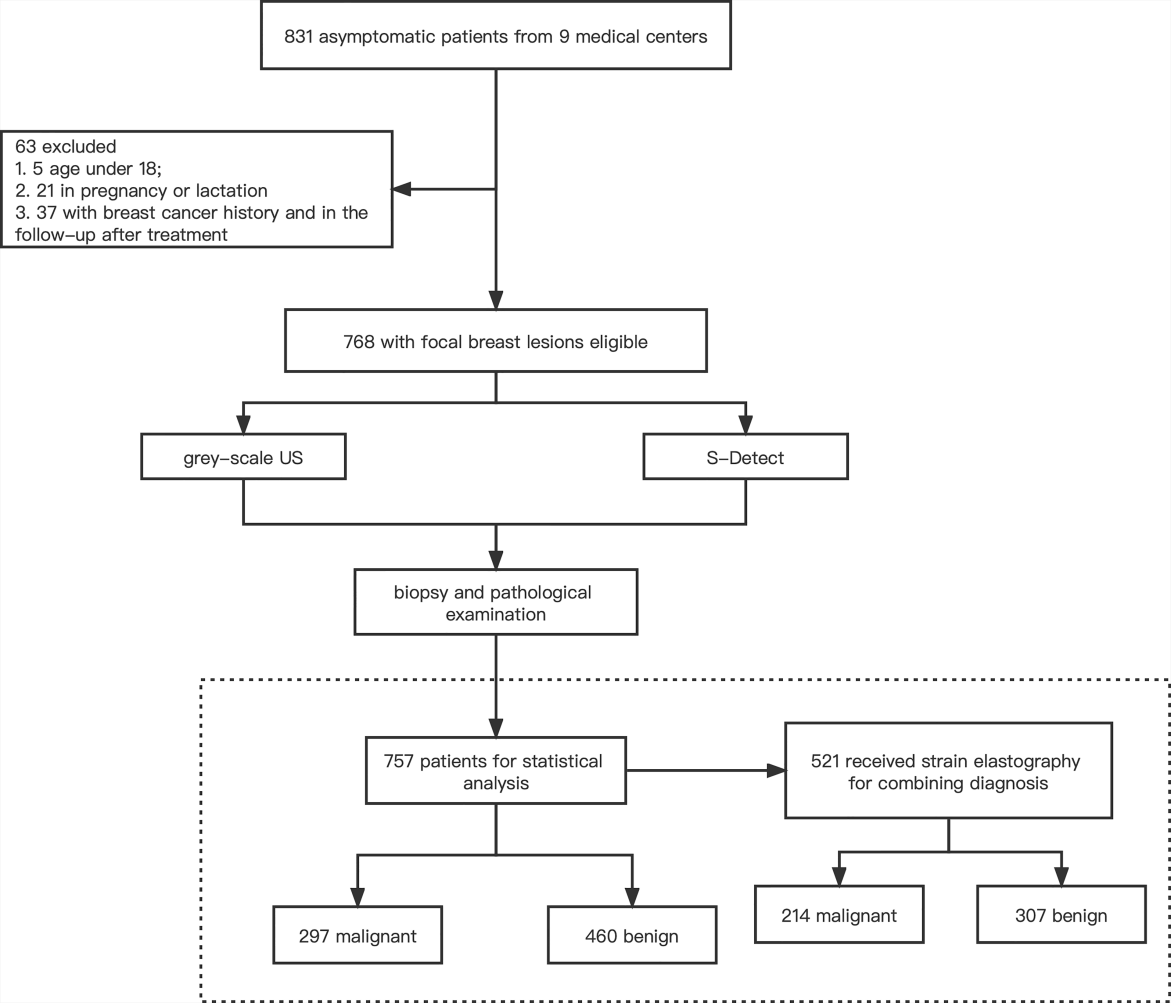
The schematic of the study flow.

A total of 831 patients participated in the study from the nine medical centers, of which 768 were eligible. Among them, 757 patients (mean age 47.5 years; median age 47.5 [15–82] years) with satisfactory imaging and pathological results were finally enrolled for statistical analysis, including 297 malignant cases and 460 benign cases, of the medical centers (**Figure 1**). The clinical characteristics and pathological results of the patients are shown in **Table 1**.

**Table 1:** Clinical information and pathological results of the patients.

Clinical information	
Age (year)	
Median (25% - 75% quartiles)	47.5 (38.00 - 56.00)
Tumor Size (cm)	
Median (25% - 75% quartiles)	1.50 (1.00 - 2.20)
Histories of benign disease	
No	654
Papillary tumors	2
Fibroma	68
Atypical hyperplasia	33
Family histories	
No	748
Yes	9
Menopause	
No	561
Yes	196
**Pathologic findings**	
Benign	460
Fibroma	205
Adenosis	173
Papillary tumors	43
Sclerosing adenopathy	9
Inflammatory lesions	19
Phyllodes tumor	11
Malignant	297
Invasive ductal carcinoma	213
Invasive lobular carcinoma	7
In situ ductal carcinoma	41
Mucinous carcinoma	8
Solid papillary carcinoma	6
Micro-papillary carcinoma	2
Encapsulated papillary carcinoma	1
Adenoid-cystic carcinoma	3
Neuroendocrine carcinoma	3
tubular carcinoma	3
Malignant phyllodes tumor	6
Lymphoma	3
Leukemia	1

### Diagnostic Performances of S-Detect™

**Figure 2:**
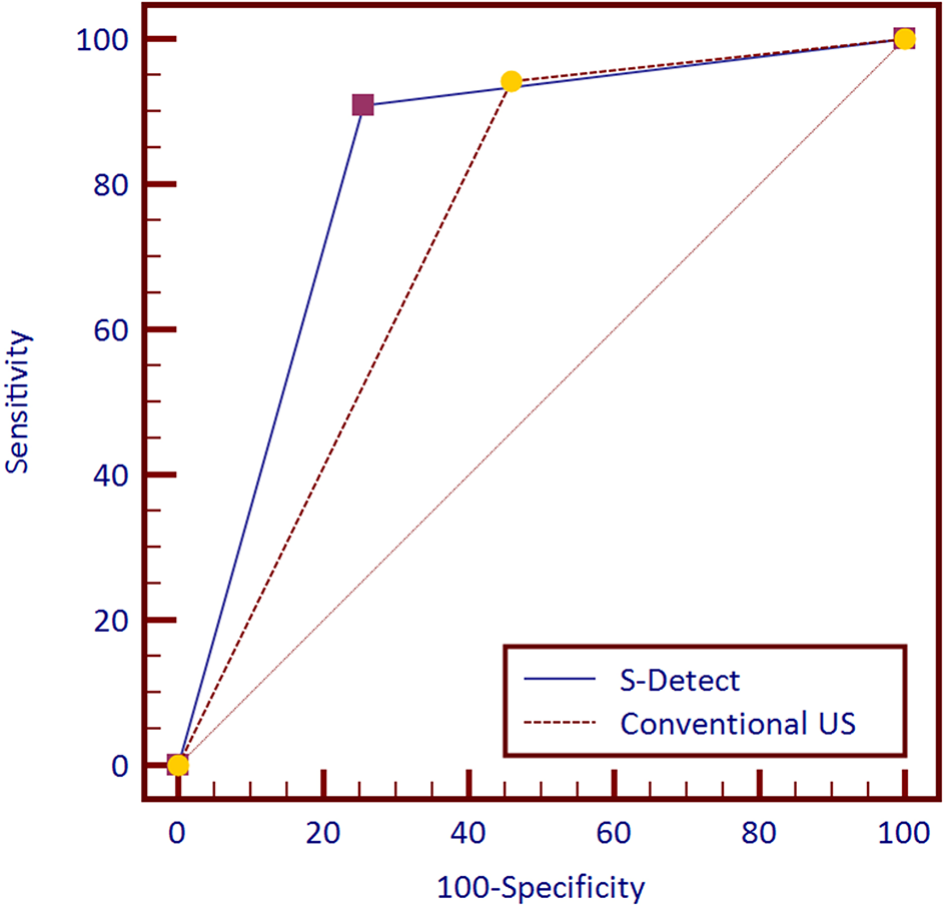
Receiver operating characteristic (ROC) curves of S-Detect and the conventional ultrasound (US) for 757 patients.

The diagnostic performances of S-Detect™ and conventional US are listed in **Table 2**. The ROC curves of the tests are illustrated in **Figure 2**. S-Detect™ was distinguished by its higher specificity and PPV than those of conventional US (specificity, 74.35% [70.10%–78.28%] vs. 54.13% [51.42%–60.29%], *p* [S-detect vs. conventional US] < 0.0001; PPV 69.59% [64.74%–74.13%] vs. 55.89% [51.42%–60.29%], *p* [S-detect vs. conventional US] < 0.0001). In the meantime, S-Detect™ possessed good sensitivity, which presented no statistical difference with that of the radiologist (91.91% [87.05%–93.92%] vs. 94.28% [90.99%–96.63%], *p* [S-Detect vs. conventional US] = 0.09). S-Detect™ also presented a high AUC value (0.83 [0.80–0.85] vs. 0.74 [0.70–0.77], *p* [S-Detect vs. conventional US] < 0.0001), suggesting its great diagnostic performance in dichotomic classification of breast lesion.

**Table 2:** The diagnostic performances of S-Detect™, conventional US, and combining diagnosis.

x	Sensitivity (%)	Specificity (%)	PPV (%)	NPV (%)	PLR	NLR	AUC
**Conventional US^1^**	94.28 (90.99–96.63)	54.13 (51.42–60.29)	55.89 (51.42–60.29)	93.61 (89.96–96.23)	2.00 (1.81–2.22)	0.11 (0.07–0.17)	0.74 (0.70–0.77)
**S-Detect^1^**	91.91 (87.05–93.92)	74.35 (70.10–78.28)	69.59 (64.74–74.13)	92.68 (89.53–95.12)	3.54 (3.02–4.16)	0.12 (0.08–0.18)	0.83 (0.80–0.85)
**Elastograohy + S-Detect^2^**	88.94 (83.99–92.78)	73.93 (68.60–78.78)	70.96 (65.17–76.28)	90.32 (85.94–93.70)	3.41 (2.81–4.15)	0.15 (0.10–0.22)	0.87 (0.84–0.90)

PPV, positive predictive value; NPV, negative predictive value; PLR, positive likelihood ratio; NLR, negative likelihood ratio; AUC, area under the receiver operating characteristics; US, ultrasound.

^1^Results for 757 patients.

^2^Results for 521 patients.

### Combined Diagnosis of S-Detect™ and Strain Elastography

**Figure 3:**
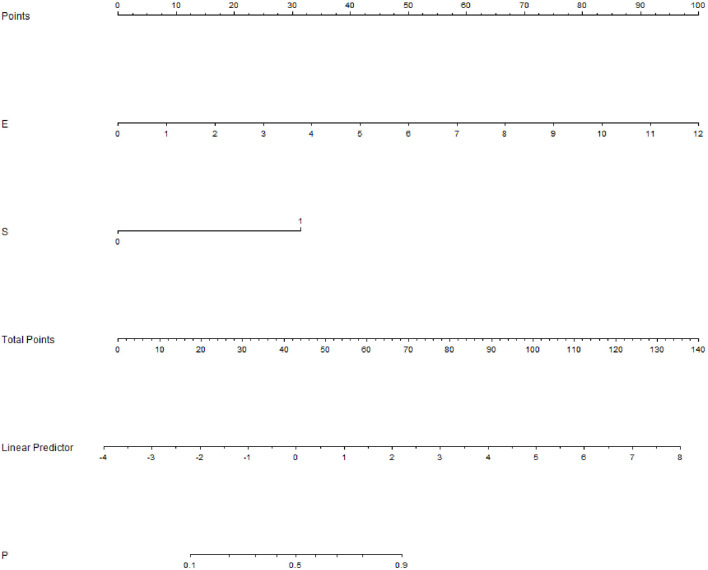
Nomogram of combined diagnosis. E, elastography; strain ratio (SR) value; S, S-Detect result; P, predictive percentage.

**Figure 4:**
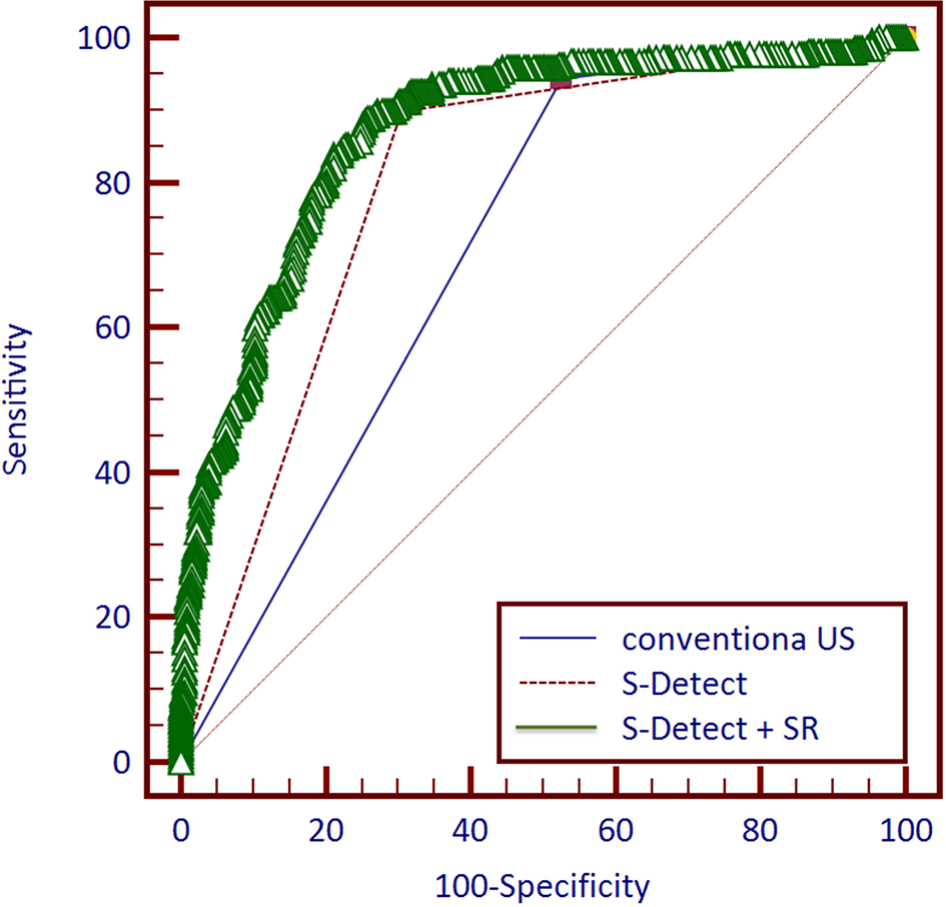
Receiver operating characteristic (ROC) curves of three methods and combined diagnosis for 521 patients.

**Figure 5:**
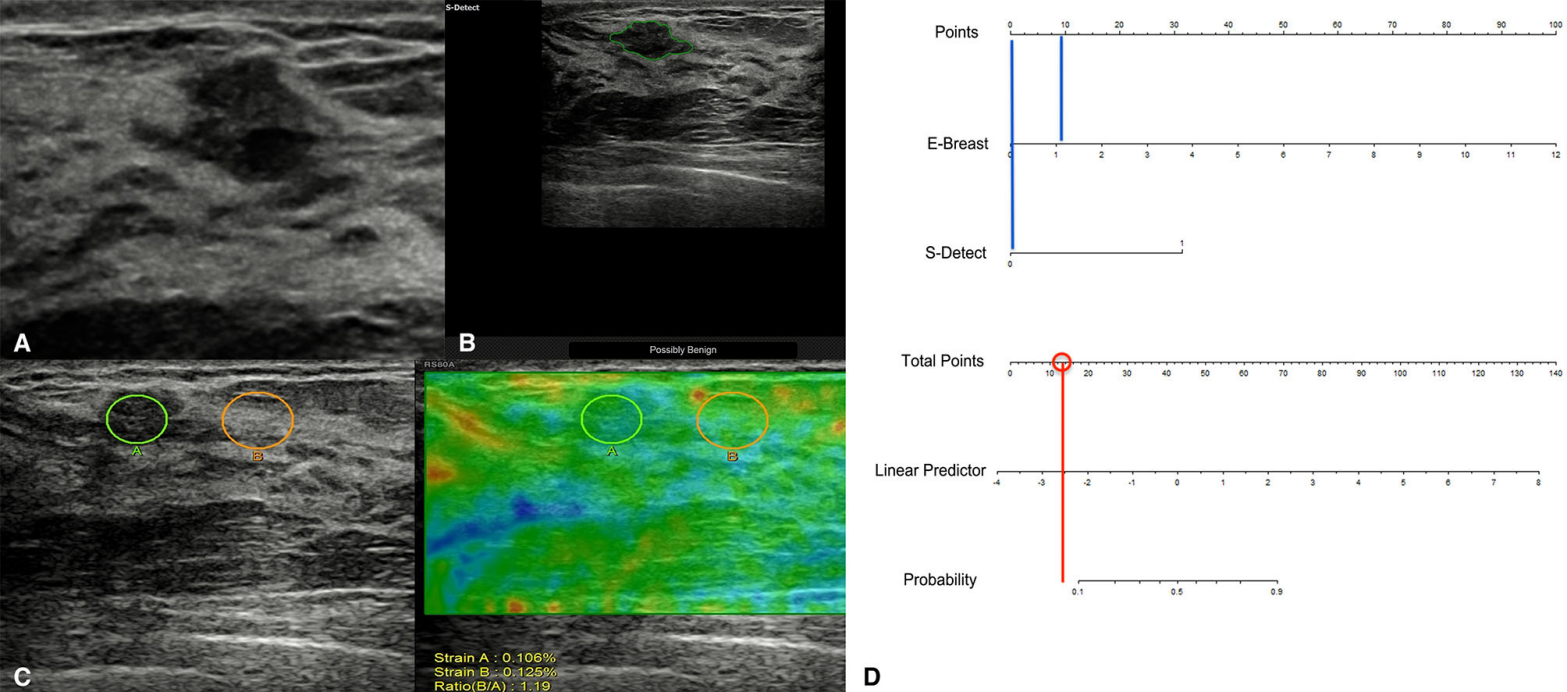
A typical case of a 45-year-old patient with a breast lesion detected and classified as BI-RADS 4a by screening ultrasound (US) (**A**; grayscale of the US). S-Detect classified it as possibly benign **(B)**, and its strain ratio (SR) was 1.19 **(C)**. According to the nomogram, the point for SR result was near 10, and the point for S-Detect was 0 (blue vertical lines), thus acquiring a total score of 10 for the lesion and a predictive percentage of less than 0.1 **(D)**. The pathological result for the lesion was adenosis.

Among the 757 enrolled patients, 521 patients also received strain elastography and had SR values. The results of the 521 patients were further used for combining diagnosis of S-Detect™ and elastography. The results of S-Detect™ and strain elastography were combined through the multiple regression method. The equation for combining diagnosis was logit(Y) = −3.80213 + 0.72155 * SR + 2.78571 * S-Detect (0/1) (Y: predictive percentage), and it was illustrated as a nomogram (**Figure 3**). The best threshold of predictive percentage for the nomogram was 0.4304. As presented in **Table 1**, under the best threshold for the combined diagnosis, the diagnostic performance was significantly enhanced after combination with an AUC value of 0.860, higher than that of S-Detect (*p* < 0.0001). The combined diagnosis also presented higher specificity and PPV (specificity, 73.93% [68.60%–78.78%] vs. 69.31% [63.78%–74.45%], *p* [combination vs. E-breast] < 0.0001; PPV 70.96% vs. 67.71%, *p* [combination vs. S-Detect] = 0.005). The ROC curves for combining results, S-Detect, and the conventional US are presented in **Figure 4**. A typical case that was misdiagnosed by the conventional US and corrected by combining diagnosis is demonstrated in **Figure 5**.

### The Differences in Diagnostic Performances Among Groups 1–4

As shown in **Table 3**, for Group 1, S-Detect presented higher specificity than the conventional US, but the sensitivity showed no difference (*p*[Sp] = 0.016; *p*[Se] = 0.25). The AUC value of S-Detect was significantly higher than the conventional US (0.89 [0.84–0.93] vs. 0.81 [0.75–0.86], *p* = 0.0013). For Group 2, S-Detect also had lower sensitivity and higher specificity than the conventional US (*p*[Sp] = 0.0001; *p*[Se] < 0.0001), and the AUC value showed no difference (0.79 [0.72–0.85] vs. 0.84 [0.77–0.89], *p* = 0.1791). For Group 3, S-Detect presented higher specificity than the conventional US, but the sensitivity showed no difference (*p*[Sp] = 0.016; *p*[Se] = 0.25). The AUC value of S-Detect was significantly higher than that of the conventional US (0.85 [0.76–0.92] vs. 0.68 [0.58–0.78], *p* = 0.0014). For Group 4, S-Detect had lower sensitivity and higher specificity than the conventional US (*p*[Sp] = 0.0001; *p*[Se] = 0.035), and it also presented a higher AUC value (0.75 [0.69–0.80] vs. 0.66 [0.60–0.72], *p* = 0.0038). In all groups, S-Detect presented higher specificity than the conventional US, and it also had higher AUC values.

**Table 3:** The diagnostic performances of S-Detect™ and conventional US in the four groups.

Group	Test	Sensitivity (%)	Specificity (%)	PPV (%)	NPV (%)	PLR	NLR	AUC
**Group 1**	S-Detect	93.75 (86.01–97.94)	83.87^2^ (77.12–89.28)	75.00 (65.34–83.12)	96.30 (91.57–98.79)	5.81 (4.04–8.36)	0.07 (0.03–0.17)	0.89^1^ (0.84–0.93)
	Conventional US	95.00 (87.69–98.62)	66.45^4^ (58.43–73.83)	59.38 (50.34–67.96)	96.26 (90.70–98.97)	2.83 (2.26–3.55)	0.08 (0.03–0.20)	0.81^3^ (0.75–0.86)
**Group 2**	S-Detect	86.30 (76.25–93.23)	80.81^2^ (71.66–88.03)	76.83 (66.20–85.44)	88.89 (80.51–94.54)	4.50 (2.97–6.81)	0.17 (0.09–0.30)	0.84^1^ (0.77–0.89)
	Conventional US	100.00^5^ (95.07–100.00)	58.59^4^ (48.24–68.40)	64.04 (54.51–72.81)	100.00 (93.84–100.00)	2.41 (1.91–3.05)	0.00	0.79^3^ (0.72–0.85)
**Group 3**	S-Detect	96.87 (83.78–99.92)	72.88 (59.73–83.64)	65.96 (50.69–79.14)	97.73 (87.98–99.94)	3.57 (2.34–5.45)	0.04 (0.01–0.30)	0.85^1^ (0.76–0.92)
	Conventional US	87.50 (71.01–96.49)	49.15 (35.89–62.50)	48.28 (34.96–61.78)	87.88 (71.80–96.60)	1.72 (1.30–2.28)	0.25 (0.10–0.66)	0.68 (0.58–0.78)
**Group 4**	S-Detect	90.18 (83.11–94.99)	59.59 (51.16–48.53)	63.13 (55.15–67.62)	88.78 (80.80–94.26)	2.23 (1.82–2.74)	0.16 (0.09–0.29)	0.75 (0.69–0.80)
	Conventional US	92.86 (86.41–96.87)	40.14 (32.15–48.53)	54.17 (46.84–94.70)	88.06 (77.82–94.70)	1.55 (1.35–1.79)	0.18 (0.09–0.36)	0.66 (0.60–0.72)

PPV, positive predictive value; NPV, negative predictive value; PLR, positive likelihood ratio; NLR, negative likelihood ratio; AUC, area under the receiver operating characteristics; US, ultrasound.

^1^AUC value of S-Detect: p (1 vs. 4) < 0.0001, p (2 vs. 4) = 0.0165, p (3 vs. 4) = 0.0157.

^2^Specificity of S-Detect: p (1 vs. 4) < 0.0001, p (2 vs. 4) = 0.0003.

^3^AUC value of the conventional US: p (1 vs. 3) = 0.0107, p (1 vs. 4) < 0.0001, p (2 vs. 3) = 0.0036, p (2 vs. 4) < 0.0001.

^4^Specificity of the conventional US: p (1 vs. 3) = 0.020, p (1 vs. 4) < 0.0001, p (2 vs. 4) = 0.0048).

^5^Sensitivity of the conventional US: p (2 vs. 3) = 0.0023, p (2 vs. 4) = 0.0048.

The diagnostic performances of S-Detect and conventional US among the groups were also compared. For the performances of S-Detect in different groups, Group 1, 2, and 3 presented a significantly higher AUC value than Group 4, and others have no differences (0.89 [0.84–0.93], 0.84 [0.77–0.89], 0.85 [0.76–0.92], and 0.75 [0.69–0.80], respectively; *p* [1 vs. 4] < 0.0001, *p* [2 vs. 4] = 0.0165, *p* [3 vs. 4] = 0.0156). Specifically, both S-Detect of Groups 1 and 2 had higher specificity than that of Group 4 (83.87% [77.12%–89.28%], 80.81% [71.66%–88.03%], and 59.59% [51.16%–48.53%], respectively; *p* [1 vs. 4] < 0.0001, *p* [2 vs. 4] = 0.0004). For the performances of conventional US in different groups, Groups 1 and 2 had a significantly higher AUC value than Groups 3 and 4 (*p* [1 vs. 3] = 0.0107, *p* [1 vs. 4] < 0.0001, *p*[2 vs. 3] = 0.0036, *p* [2 vs. 4] < 0.0001). The comparisons in sensitivity, specificity, and AUC values among the four groups are shown in **Table 3**.

## Discussion

US has enjoyed great popularity in China as one of the most essential imaging methods for detecting breast cancer. It usually presents very high sensitivity but relatively low specificity (8). The low specificity and PPV of breast US causing high recall rate and unnecessary biopsies in breast screening have been major problems in the clinical utilization of US (33, 34). Efforts have been made to conquer this problem by applying other US modalities in addition to the grayscale US. In this multicenter study, we investigated the value of CAD and elastography in strengthening the diagnostic performance of US for the asymptomatic breast lesions detected by opportunistic screening US. The recruited patients in this study underwent breast US screening and were recalled for the diagnostic US. With the addition of S-Detect and the combination of S-Detect™ and elastography, the performance of US can be significantly enhanced, especially the specificity and PPV. These US techniques are promising in further clinical promotion for breast imaging, as an important adjunct to the routine US in detecting and diagnosing breast cancer.

In recent years, several self-developed or commercialized CAD systems for breast US based on DL methods have been developed and shown good performance in the detection, segmentation, and diagnosis of breast lesions (35, 36). S-Detect™ is one of the DL-based CAD systems, constructed on convolutional neural networks (CNN) and trained by a large number of images of breast masses. Free from impact from handcrafted features, the CAD system can make segmentation and dichotomic classification of breast lesions automatically. According to previous studies from 2016 to 2019 about S-Detect™, the commercial CAD system presented outstanding accuracy and specificity in classifying breast lesions, thus holding potentials in enhancing the diagnostic performance of human readers (17–20). In this study, the higher AUC value and specificity of S-Detect™ compared with the conventional US were also verified (AUC, 0.799; specificity, 0.695), similar to previous reports, which also revealed an increment in specificity (0.78–0.90) and AUC value (0.80–0.92). The sensitivity was still maintained at a relatively high level, without statistical decrease. With the use of S-Detect™, unnecessary biopsies can be effectively reduced for those asymptomatic screening breast lesions.

In a common clinical situation, radiologists make a diagnosis of breast lesions by integrating clinical information and comprehensive imaging information. For those patients with typical clinical manifestations, such as severe pain, nipple discharge, and fast-growing nodules, the lesions might be upgraded by radiologists. In terms of the asymptomatic US screening-detected breast lesions, based on the results of our study, we can safely conclude that S-Detect™ is a reliable method in downgrading possibly benign lesions and avoiding unnecessary biopsies, which can be further applied in US screening.

The role of elastography has been established in recent years as an essential assisting method for breast US. A combination of elastography and the conventional US could benefit the diagnosis of breast lesions by improving specificity without lowering sensitivity (37, 38). In this study, we combined the CAD technique and elastography to further enhance the diagnostic performance of US for asymptomatic breast nodules. The combined diagnosis presented higher accuracy and specificity, compared with a single use of S-Detect™ and the conventional US, without lowering sensitivity. Moreover, both S-Detect™ and strain elastography (E-breast) can make objective assessments of breast lesions, independent of the conventional US diagnosis process. The two methods can play a complementary role for each other in collecting diagnostic information of breast nodules. In view of the results of this study, the combination of elastography and S-Detect™ has a significant clinical value in improving the specificity and overall performance of US in classifying the asymptomatic breast lesions, which in turn can reduce unnecessary biopsies for those US-screening-detected lesions. The easy access of the two built-in US techniques may also further facilitate their integration into US operating routine, without increasing workload.

We also compared the diagnostic performances in different groups of medical centers in this study. Based on the results, we can conclude that in most cases, S-Detect presents higher specificity and overall performances than the conventional US, which further validates its feasibility in diagnosing breast lesions. Additionally, due to its considerable accuracy in different regions of China, S-Detect’s stability can be recognized in this multicenter study, and it is promising for further clinical promotion. However, significant differences in the AUC values of S-Detect of different regions were detected between groups. These centers also had different performances of conventional US. For the centers with better performances of human readers, S-Detect also exhibited higher diagnostic accuracy. This issue was not previously reported in other single-center studies of S-Detect. It might be suggested that the training of US operators in the application of CAD is still essential. In the medical centers with better-trained US operators, more standard acquisition of US imaging for the CAD analysis can be realized, thus realizing the better performance of S-Detect.

There existed several limitations in this study. Firstly, the cases included are suspicious lesions found by US. The proportion of invasive ductal carcinoma is relatively high, and further studies are required to evaluate the value of the methods in diagnosing *in situ* ductal carcinoma. Also, we did not take the results of mammography into consideration in this study. The clinical information of the patients could also be included in further studies to construct a more comprehensive diagnostic model.

## Conclusion

S-Detect™, a CAD system for breast US, presented a good diagnostic performance in classifying asymptomatic breast lesions detected by opportunistic screening, with a higher overall AUC value and specificity than the conventional US. After the results and strain elastography were combined, both of which could provide objective imaging information for breast nodules, the overall performance and specificity could be further improved. Characterized by the aid for screening US in enhancing diagnostic efficacy and reducing unnecessary biopsies, S-Detect™ and its combination with elastography can be further utilized clinically.

## Data Availability Statement

The data analyzed in this study is subject to the following licenses/restrictions: the datasets are available from the corresponding author on reasonable request. Requests to access these datasets should be directed to zqlpumch@126.com.

## Ethics Statement

The studies involving human participants were reviewed and approved by the Institutional review board of Peking Union Medical College Hospital. The patients/participants provided their written informed consent to participate in this study.

## Author Contributions

QlZ, YJ, CZ, and MX contributed to the conception and design of the study. MX, XY, JD, LC, FG, MW, LL, QC, WC, JG, QL, QZh, JL, and QlZ organized the database. CZ and MX performed the statistical analysis. CZ wrote the first draft of the manuscript. LM and MX wrote sections of the manuscript. QlZ revised the manuscript. All authors contributed to manuscript revision and read and approved the submitted version.

## Funding

This work was funded by the CAMS Innovation Fund for Medical Sciences (CIFMS) (2020-I2M-C&T-B-033).

## Conflict of Interest

The authors declare that the research was conducted in the absence of any commercial or financial relationships that could be construed as a potential conflict of interest.

## Publisher’s Note

All claims expressed in this article are solely those of the authors and do not necessarily represent those of their affiliated organizations, or those of the publisher, the editors and the reviewers. Any product that may be evaluated in this article, or claim that may be made by its manufacturer, is not guaranteed or endorsed by the publisher.

## Abbreviations

US, ultrasound (US); BI-RADS, Breast Imaging Reporting, and Data System; PPV, positive predictive value; CAD, computer-aided diagnosis; DL, deep learning; SR, strain ratio; PLR, positive likelihood ratio; NLR, negative likelihood ratio; NPV, negative predictive value; ROC, receiver operating characteristic; AUC, area under the receiver operating characteristics curve; CNN, convolutional neural networks.
